# HIV/AIDS, growth and poverty in KwaZulu-Natal and South Africa: an integrated survey, demographic and economy-wide analysis

**DOI:** 10.1186/1758-2652-12-18

**Published:** 2009-09-16

**Authors:** James Thurlow, Jeff Gow, Gavin George

**Affiliations:** 1 International Food Policy Research Institute, Washington DC, USA; 2 Department of Economics, University of Copenhagen, Copenhagen, Denmark; 3 School of Accounting, Economics and Finance, University of Southern Queensland, Toowoomba, Australia; 4 Health Economics and HIV/AIDS Research Division (HEARD), University of KwaZulu-Natal, Durban, South Africa; 5 HEARD, University of KwaZulu-Natal, Durban, South Africa

## Abstract

**Background:**

This paper estimates the economic impact of HIV/AIDS on the KwaZulu-Natal province and the rest of South Africa.

**Methods:**

We extended previous studies by employing: an integrated analytical framework that combined firm surveys of workers' HIV prevalence by sector and occupation; a demographic model that produced both population and workforce projections; and a regionalized economy-wide model linked to a survey-based micro-simulation module. This framework permits a full macro-microeconomic assessment.

**Results:**

Results indicate that HIV/AIDS greatly reduces annual economic growth, mainly by lowering the long-run rate of technical change. However, impacts on income poverty are small, and inequality is reduced by HIV/AIDS. This is because high unemployment among low-income households minimises the economic costs of increased mortality. By contrast, slower economic growth hurts higher income households despite lower HIV prevalence.

**Conclusion:**

We conclude that the increase in economic growth that results from addressing HIV/AIDS is sufficient to offset the population pressure placed on income poverty. Moreover, incentives to mitigate HIV/AIDS lie not only with poorer infected households, but also with uninfected higher income households.

Our findings reveal the substantial burden that HIV/AIDS places on future economic development in KwaZulu-Natal and South Africa, and confirms the need for policies to curb the economic costs of the pandemic.

## Background

South Africa has one of the highest HIV prevalence rates in the world, and KwaZulu-Natal (KZN) is its worst afflicted province. Recent estimates indicate that 26.4% of KZN's working age population is HIV positive, compared to 15.9% in the rest of the country [1]. Unemployment and income poverty in the province are also much higher than the national average. More than a third of KZN's population live below the US$2 a day poverty line and two-fifths of the workforce is unemployed [2,3].

Long-term trends in KZN are equally bleak. Recent evidence indicates that economic growth continues to lag behind the rest of the country, and that poverty is rising faster than in other provinces. Therefore, a key challenge for reviving economic development in South Africa, and in KZN in particular, is to understand the constraints imposed by HIV/AIDS on future economic growth and poverty reduction.

Numerous studies estimate the micro-level impacts of the pandemic (see [4] for an overview). These confirm the severe detrimental effects imposed on infected individuals and their households. However, while household-based studies are better able to capture detailed non-economic impacts, they typically overlook systemic or economy-wide shocks from HIV/AIDS. These can have indirect or "second-round" consequences for both infected and uninfected population groups. Some studies have assessed the broader implications of HIV/AIDS for economic growth and employment in South Africa (see, for example, [5]).

However, these macroeconomic studies were conducted when detailed micro-level data on prevalence rates for different sectors and occupations were not yet available. This information on HIV prevalence among firms and workers permits a more accurate assessment of the consequences of the pandemic. Moreover, the availability of these micro-level estimates allows for more integrated approaches to measuring socioeconomic outcomes.

In this paper, we estimate the growth and distributional impacts of HIV/AIDS on KZN and the rest of South Africa (Other SA). First, we conducted a firm survey in four of KZN's largest sectors. Second, information on workers' HIV prevalence rates from the survey was used to calibrate an occupation-focused demographic model. Finally, the demographic projections were imposed on a regionalized dynamic computable general equilibrium (DCGE) model linked to a household survey-based micro-simulation model. This integrated macro-microeconomic framework permits a more robust empirically based assessment of the impacts of HIV/AIDS.

The next section briefly describes the survey and demographic projections, as well as outlining the methodology, paying particular attention to the links between the demographic and DCGE models. The following section discusses the DCGE model's results and their implications for future socioeconomic development in South Africa. The final section summarizes the findings.

## Methods

### Demographic impacts of HIV/AIDS in South Africa

The first stage of our analysis combines two demographic models. For a detailed description of the demographic model and projections, see [6]. The first model estimates provincial population projections for different population groups. Based on these results, the second model estimates workforce projections by occupational groups. The parameters of the second demographic model are calibrated to HIV prevalence rates from a firm-level survey of workers. This section describes the population projections and HIV prevalence profile, followed by the firm survey and workforce projections.

### Population projections

The provincial version of the ASSA-2003 model from the Actuarial Society of South Africa [1] was used to estimate overall population projections for KZN and Other SA. The model produces annual population estimates with and without the effects of HIV/AIDS for the period of 1985 to 2025. The ASSA model disaggregates the total population by province, gender, racial groups (African, Asian, coloured and white) and one-year age intervals. HIV in the model is spread via heterosexual sexual activity among adults, who are divided into risk groups according to sexual behaviour. The calibration of the model is based on epidemiological and medical research, population census data, and HIV prevalence data from antenatal clinic surveys and mortality statistics. Table 1 provides a profile of HIV prevalence for the year 2002, which is the base year for our economic analysis in later sections.

**Table 1:** HIV prevalence among working age adults, 2002

Population group	Gender	Age cohort	Population (millions)		HIV prevalence (%)	
			
			Other SA	KZN	Other SA	KZN
National	Both	All	35,252	9,250	8.7	13.4
						
Africans	Male	20-34	3,695	990	19.6	30.6
		35-49	2,241	507	24.8	41.3
		50-64	961	236	11.9	21.4
						
	Female	20-34	3,820	1,088	29.8	43.3
		35-49	2,430	655	16.2	27.3
		50-64	1,141	325	1.6	3.0
						
Other races	Male	20-34	995	172	1.8	1.5
		35-49	875	160	2.3	2.2
		50-64	521	108	0.6	0.7
						
	Female	20-34	1,011	174	3.9	3.6
		35-49	924	170	3.2	3.0
		50-64	571	120	0.4	0.4

Source: Own calculations using estimates from [1] and [6].

Other SA: Rest of South Africa

KZN: KwaZulu-Natal

HIV prevalence is concentrated among working age Africans, especially younger females (20 to 34 years) and slightly older males (35 to 49 years). By contrast, prevalence for the other racial groups is considerably lower for all age cohorts. Moreover, prevalence among Africans is heavily concentrated within KZN - a pattern that does not exist for other races. Given the large African and KZN population, it is clear that this province and population group forms the epicentre of South Africa's HIV pandemic. The effects of this concentration are evident in the population projections from the ASSA model (Table 2).

**Table 2:** Demographic projections, 2002-2025

		Population (millions)		Prevalence rate (%)	AIDS-sick rate (%)
				
		No AIDS	AIDS		
KZN	1990	3.54	3.54	0.39	0.00
	1995	4.13	4.12	7.35	0.11
	2000	4.64	4.57	23.18	1.28
	2005	5.25	4.87	27.95	3.49
	2010	5.90	5.06	27.59	3.80
	2015	6.52	5.24	26.85	3.79
	2020	7.16	5.43	26.49	3.70
	2025	7.70	5.52	26.17	3.70
Other SA	1990	13.51	13.51	0.16	0.00
	1995	16.16	16.16	3.42	0.05
	2000	18.53	18.40	13.16	0.63
	2005	20.96	20.15	18.06	1.92
	2010	23.27	21.35	18.99	2.26
	2015	25.22	22.09	18.74	2.50
	2020	27.10	22.71	18.18	2.50
	2025	28.73	23.12	17.78	2.43

Source: Own calculations using estimates from [1].

The long-term implications of HIV/AIDS for population growth are pronounced. Without its effects, South Africa's adult population is predicted to have reached 36.4 million by 2025. AIDS deaths reduce this adult population by 7.8 million people, which is more than a quarter of the expected population in 2025. The predicted loss of life in KZN is even more staggering, with two-fifths of the adult population having died from HIV/AIDS by 2025. The pandemic is, however, expected to peak around 2010, with HIV prevalence rates beginning to fall and AIDS-related sickness and death declining after 2020. Despite "turning the corner", the scale of the pandemic and its concentration among working age adults will have grave implications for South Africa's workforce.

### Firm survey and workforce projections

Previous studies relied on population projections to estimate the economic consequences of HIV/AIDS in South Africa. As part of our study, an AIDS Projection Model (APM) was developed to estimate size of the workforce with and without HIV/AIDS [6]. The model distinguishes between three occupations (managers, skilled workers and labourers), genders, two racial groups (African and other races), and three age cohorts (20-34, 35-49 and 50-64). The APM is a demographic model and so cannot predict changes in workforce composition (i.e., shifts in sectoral employment patterns driven by economic forces). This is the domain of the economy-wide model.

However, the APM does combine the ASSA model's population projections with HIV test data from a firm survey to predict the impact of HIV/AIDS on the size of the workforce for different occupational groups. The changing sectoral composition of employment is endogenously determined by the DCGE model. It therefore provides the critical link between the population projections and the economic analysis in the next section.

The calibration of the APM was based on the firm survey data collected during our study. Anonymous HIV tests were conducted for 15 companies in four economic sectors: agriculture, manufacturing, tourism and transport. The 15 companies were surveyed over three years: two in 2005, 11 in 2006, and two in 2007. For convenience, we treated all survey results as reflecting HIV prevalence in 2006. These are key sectors of the South African economy. Together, they comprise 59.1% and 44.7% of KZN and South Africa's gross domestic product respectively, and 55.8% and 49.1% of labour employment.

A total of 6197 workers were tested, but only 4464 questionnaires were completed successfully. The sample had an overall HIV prevalence rate of 16.7%, with a 95% confidence interval of ± 1.1%. Table 3 presents the prevalence rates for male African workers by sector and occupation. The survey-based estimates of HIV prevalence were "smoothed" to account for wider confidence intervals for specific subgroups of the sample (see [6]).

**Table 3:** HIV prevalence rates for male Africans by occupation, 2002

Sector	Age cohort	Occupation groups		
		
		Managers	Skilled	Labourers
Agriculture	20-34	33.9	29.8	35.0
	35-49	37.8	32.6	38.2
	50-64	16.8	16.3	19.1
				
Manufacturing	20-34	22.2	24.9	31.1
	35-49	24.7	27.2	33.9
	50-64	0.0	14.0	17.6
				
Tourism	20-34	29.9	34.1	37.6
	35-49	33.8	37.3	40.9
	50-64	0.0	18.4	20.0
				
Transport	20-34	13.4	20.5	32.5
	35-49	14.3	22.4	35.1
	50-64	7.5	11.3	17.9

Source: Own calculations using estimates from [6].

The survey reveals considerable heterogeneity across workers. Prevalence rates are typically highest for labourers (i.e., unskilled workers) within the agriculture and tourism sectors. They are lowest for managers and professionals, with the exception of agriculture, where prevalence rates are similar for all three occupational groups. Prevalence is significantly higher for the middle-age cohort, which is consistent with observed national trends.

The survey clearly indicates that it is inappropriate to make broad generalizations about the sectoral and occupational trends of HIV prevalence. Therefore, the inclusion of an empirically calibrated APM that produces occupation-based workforce projections greatly enhances the accuracy of our economic analysis *vis-à-vis *previous studies. It also provides a crucial link between the economic growth impacts of HIV/AIDS and its effects on employment, poverty and inequality. The next section describes how these demographic projections are incorporated within the economic modelling.

### Estimating the economic impacts of HIV/AIDS

HIV/AIDS affects economic growth and poverty via various impact channels. At the household level, a wide range of factors influence poverty, including: vulnerability from deteriorating livelihoods; heightened stigmatisation and a fragmentation of social networks; and lower investments in human capital and nutrition. These household-level effects need to be aggregated in order to estimate the overall impact of the pandemic.

Moreover, while households are directly affected by HIV/AIDS, there are also broader implications for the economy as a whole. In our macro-microeconomic assessment, we account for not only households, but also other actors or institutions, such as firms, markets and government. However, broadening our analysis necessarily excludes some difficult-to-measure household-level impacts. Therefore, given our focus on economic growth, we concentrate on the income dimensions of poverty. Ultimately we identify five main impact channels for HIV/AIDS: population growth; labour supply; labour productivity; total factor productivity; and savings and investment. This section describes how these impact channels are captured in the economy-wide model

### Simplified general equilibrium model

Additional file 1 presents the equations of a simple closed-economy computable general equilibrium (CGE) model that illustrate how HIV/AIDS affects economic outcomes in our analysis. The model is recursive dynamic and so can be separated into a static "within-period" component, where producers and consumers maximize profits and utility, and a dynamic "between-period" component, where the model is updated based on the demographic model and previous period results to reflect changes in population, labour supply, and capital and technology accumulation.

In the static component of the model, producers in each sector *s *and region *r *(i.e., KZN and Other SA) produce a level of output *Q *in time period *t *by employing the factors of production *F *under constant returns to scale (exogenous productivity *α*) and fixed production technologies (fixed factor shares δ) (eq. [1]). Profit maximization implies that factor payments *W *are equal to average production revenues (eq. [2]). Labour supply *L *and capital supply *K *are fixed within a given time period, implying full employment of factor resources. Labour market equilibrium is defined at the regional level so that labour is mobile across sectors, but wages vary by region (eq. [6]). National capital market equilibrium implies that capital is mobile across both sectors and regions and earns a national rental rate (i.e., regional capital returns are equalized) (eq. [7]).

Factor incomes are distributed to households in each region using fixed income shares based on households' initial factor endowments (eq. [3]). Total household incomes *Y *are then either saved (based on marginal propensities to save υ) or spent on consumption *C *(according to marginal budget shares β) (eq. [4]). Consumption spending includes a "subsistence" component λ that is independent of income and determined by household populations *H*. Savings are collected in a national savings pool and used to finance investment demand *I *(i.e., savings-driven investment closure) (eq. [5]).

Nell empirically tests the causality between national savings and investment in South Africa, and confirms the appropriateness of a savings-driven investment closure [7]. Finally, a single price *P *equilibrates national product markets, thus avoiding having to model inter-regional trade flows (eq. [8]). A consumer price index weighted by the aggregate household consumption basket is the model's *numéraire*.

The model's variables and parameters are calibrated to observed data from a provincial social accounting matrix that captures the initial equilibrium structure of the KZN and Other SA economies in 2002. A social accounting matrix is a consistent database capturing all monetary flows in an economy in a given year. It contains information on the production technologies and demand structures of detailed sectors, regions and households, as well as government revenues and expenditures and foreign receipts and payments. Various datasets were used to build the 2002 provincial social accounting matrix for South Africa, including: national accounts; the 2000 Income and Expenditure Survey; the 2002 Labour Force Survey; and the South African Standard Industrial Database [8].

The income and expenditure data was reconciled using cross-entropy estimation [9]. Parameters are then adjusted over time to reflect demographic and economic changes and the model is re-solved or a series of new equilibriums for the period of 2002 to 2015. Two simulations are conducted - "AIDS" and "No AIDS" - and the difference in the variables' final values is interpreted as the impact of HIV/AIDS. For more information on the social accounting matrix, see [10].

### Dynamic impacts of HIV/AIDS

Between periods, household populations *H *increase at rates determined by the demographic model (eq. [9]). Individual-level population projections *DH *are estimated for each region *r*, population group *p*, gender *g *and age cohort *a*, and then compared to predicted population levels *dh *in the base year 2002. The 2002 year is an appropriate base for both the "AIDS" and "No AIDS" scenario since it predates most of the main effects of HIV/AIDS on South Africa's working population. This ratio is multiplied by the observed demographic composition *sh *of each household group *h *in the CGE model to arrive at household-level population time series for 2002 to 2025.

Demographic compositions are drawn from the re-weighted 2000 Income and Expenditure Survey [11]. Similarly, labour supplies are based on demographic projections for occupation-based skill groups (eq. [10]). The factor subscript *f *is a composite for a worker's population group *p*, gender *g*, and occupation *o*. Population and labour supply in the DCGE model draws directly on the demographic projections *DH *and *DL *to capture the first two impact channels of HIV/AIDS. By increasing mortality, the pandemic reduces consumer demand and the productive capacity of the economy, both of which are likely to have adverse impacts on economic growth.

The third impact channel is the effect of morbidity on workers' productivity. This is captured in (eq. [11]), where the labour productivity growth rate ε depends on the exogenous productivity growth μ adjusted for share of the population that is HIV positive *DP *or AIDS sick *DA *(i.e., suffering from full-blown AIDS). Selected values of DP and DA for the entire population are given in the final two columns of Table 2.

In the "No AIDS" scenario, *DP *and *DA *are zero and labour productivity grows at *μ*. This growth rate is lower in the "AIDS" scenario because we assume that HIV-positive workers are half as productive as uninfected workers and that AIDS-sick workers are a fifth as productive. This is caused by lower on-the-job productivity and more days absent from work. Although the prevalence rates are estimated by the demographic model, the impact of morbidity on worker productivity must be assumed, because there are few empirical studies estimating workers' productivity losses from HIV/AIDS.

Given the findings of the impact of HIV/AIDS on tea pickers in Kenya, our assumptions may be an upper bound estimate of productivity losses. However, as seen in the next section, this impact channel is found to contribute the least to the overall economic impact of HIV/AIDS [12].

The fourth impact channel is the reduction in total factor productivity (TFP) caused by *systemic *shocks to the economy (eq. [12]). For example, AIDS morbidity and mortality reduces the productivity of uninfected workers by disrupting the production process. Moreover, the death of education and health professionals has long-term detrimental effects on the entire economic system. Unfortunately, this impact channel cannot be calibrated using the firm survey or demographic model. Thus, given the lack of evidence, we assume that AIDS reduces annual TFP growth φ by around 0.5% per year. This is similar to the TFP losses used in other studies of South Africa and Botswana [5,13].

The final impact channel is the adverse effect on savings and investment (see [14]). HIV/AIDS increases households' healthcare spending and lowers spending on other products, such as food, shelter and clothing. As a coping strategy, households draw on assets or savings. Accordingly, it is assumed that an infected households' share of disposable income spent on health care increases by 5% and savings rates are reduced by the same amount (i.e., β and υ in eq. [4]). This lowers the overall level of savings and investment (eq. [5]).

Investment from the previous period is then converted into new capital stocks using a fixed capital price κ (eq. [13]). This is added to previous capital stocks after applying a fixed rate of depreciation π. New capital is allocated to regions and sectors endogenously in order to equalize capital returns. The model therefore endogenously determines the national rate of capital accumulation and supply of capital *K*. If HIV/AIDS reduces national income, then it lowers the level of savings and funds that can be invested in the economy, thus reducing the rate of capital accumulation and further reducing long-term economic growth.

### Extensions to the full model

The simplified model illustrates how HIV/AIDS affects economic outcomes in our analysis. However, the full model drops certain assumptions. The full DCGE model is an extended version of the national model described in [10]. Constant elasticity of substitution (CES) production functions allow factor substitution based on relative factor prices (i.e., δ is no longer fixed).

The model identifies 25 sectors in KZN and Other SA. The 25 sectors are mapped onto the four sectors in the firm survey. Most of the sectors in the DCGE model are in manufacturing, but we assume similar prevalence rates for mining. Similarly, we assign the tourism sector prevalence rates to the retail trade sector, and the transport sector prevalence rates to the remaining service sectors in the DCGE model. Intermediate demand in each sector (excluded in the simple model) is determined by fixed technology coefficients.

Regional labour markets are further segmented across race, gender and three occupation-based skill categories. A nested demand system places skill levels above gender and age groups. All factors are assumed fully employed, and capital is immobile across sectors. New capital from past investment is allocated to regions and/or sectors according to profit rate differentials under a "putty-clay" specification (see [15]).

The full model still assumes national product markets for most commodities. However, international trade is captured by allowing production and consumption to shift imperfectly between domestic and foreign markets depending on the relative prices of imports, exports and domestic goods. South Africa is a small country and so world prices are fixed and the current account balance is maintained by a flexible real exchange rate (i.e., price index of tradable to non-tradeable goods). Production and trade elasticities are econometrically estimated.

Households maximise a Stone-Geary utility function such that a linear expenditure system determines consumption and permits non-unitary income elasticities. The latter are drawn from [16]. Households are disaggregated across KZN and Other SA, the racial group of the household head (i.e., African and other), and across 14 income groups (i.e., 10 deciles with the top decile separated into five income groups). These household groups pay taxes to government, based on fixed direct and indirect tax rates. Tax revenues finance exogenous recurrent spending resulting in an endogenous fiscal deficit.

Finally, the model includes a micro-simulation module in which each household in the 2000 Income and Expenditure Survey [11] is linked to its corresponding representative household in the DCGE model. Changes in households' real consumption spending on each commodity are passed down from the DCGE model to the household survey, where total per capita consumption and poverty measures are recalculated.

In summary, the full DCGE model captures the detailed sectoral and labour market structure of South Africa's economy as well as the linkages between production, employment and household incomes. Moreover, the results from the firm survey and demographic model are explicitly integrated within the economic analysis. Although not exhaustive, the five main impact channels captured by the DCGE model provide a reasonable approximation of the consequences of HIV/AIDS for growth, poverty and inequality.

## Results and discussion

Two simulations are conducted to estimate the impact of HIV/AIDS during the period of 2002 to 2025. The "AIDS" scenario captures the current growth path of KZN and South Africa, drawing on the demographic projections for population and labour supply, and observed trends for TFP and labour productivity growth. Demographic projections provide time-series estimates for *DH *(eq. [9]), *DL *(eq. [10]), and *DP *and *DM *(eq. [11]. Observed trends for 1990 to 2007 provide estimates of *μ *(eq. [11]), *φ *(eq. [12]) and *π *and *κ *(eq. [13]). Together these parameters define the exogenous dynamic component of the DCGE model. Static component parameters and behavioural elasticities are either econometrically estimated or drawn from the 2002 social accounting matrix. Then, in the hypothetical "No AIDS" scenario, we adjust the demographic projections to capture the higher population, labour supply and productivity growth rates in the absence of HIV/AIDS. In this section, we compare the results from these two simulations.

### Growth and employment

Tables 4 and 5 present the growth and employment results from the DCGE model. Given the demographic projections, HIV/AIDS reduces KZN's overall population growth rate from an average 1.85% from 2002 to 2025 in the "No AIDS" scenario to 0.79% in the "AIDS" scenario. This is larger than the decline in the population growth rate for Other SA due to the province's higher HIV prevalence. Similarly, declines in the African population are substantially larger than for other races due to higher prevalence among Africans.

**Table 4:** Growth and poverty results, 2002-2025

	KwaZulu-Natal (KZN)			Other South Africa (Other SA)		
	
	Initial, 2002	Annual growth (%)		Initial, 2002	Annual growth (%)	
				
		AIDS	No AIDS		AIDS	No AIDS
GDP (R billions)	171	2.84	4.44	872	3.04	4.46
GDP per capita (R)	18,464	2.03	2.54	24,723	2.23	2.88
Population (millions)	9,250	0.79	1.85	35,252	0.79	1.54
African	7,999	0.93	2.08	28,045	0.94	1.80
Other	1,252	-0.23	-0.03	7,207	0.17	0.37
Dependency ratio (pop/employment)	4.86	5.05	4.98	4.41	4.40	4.31
African households	5.57	5.62	5.38	4.94	4.82	4.60
Other households	2.69	2.73	2.82	3.12	3.13	3.21
Total factor productivity	-	0.03	0.60	-	-0.04	0.50
Household savings rate (%)	1.76	1.40	3.51	0.50	0.40	1.00
Health spending share of income (%)	13.55	20.87	14.33	14.02	21.44	14.90
Poverty rates (%)						
Incidence of poverty (P0)	36.66	19.46	20.00	24.83	10.50	9.51
Depth of poverty (P1)	14.73	6.02	6.20	9.40	3.46	3.15
Severity of poverty (P2)	7.71	2.69	2.77	4.91	1.74	1.60
Number of poor people (thousands)	3,391	2,157	2,819	8,752	4,438	4,759
Number of AIDS deaths (thousands)	-	3,011	0	-	7,793	0

Source: Provincial DCGE model results.

Notes: Poverty is based on US$2 a day poverty line (R161 per adult equivalent per month in 2000 prices).

R: South African rands

**Table 5:** Labour market results, 2002-2025

	KwaZulu-Natal (KZN)			Other South Africa (Other SA)		
	
	Initial, 2002	Annual growth (%)		Initial, 2002	Annual growth (%)	
				
		AIDS	No AIDS		AIDS	No AIDS
Employment (1000s)	1,902	0.63	1.75	7,988	0.81	1.64
African	1,436	0.90	2.24	5,677	1.05	2.11
Skilled	184	0.87	1.73	679	1.01	1.67
Semi-skilled	718	0.99	2.23	2,844	1.06	2.04
Low skilled	534	0.78	2.43	2,154	1.05	2.33
Other	466	-0.31	-0.24	2,311	0.15	0.24
						
Labour productivity	-	1.80	1.92	-	1.80	1.88
African	-	1.80	2.02	-	1.80	1.95
Skilled	-	1.80	1.93	-	1.80	1.89
Semi-skilled	-	1.80	2.02	-	1.80	1.96
Low skilled	-	1.80	2.10	-	1.80	2.00
Other	-	1.80	1.82	-	1.80	1.82
						
Wages (Rands)	75,511	3.09	4.05	96,054	2.94	3.93
African	59,219	2.48	2.88	91,944	2.67	3.33
Skilled	64,824	2.53	3.24	120,083	2.76	3.63
Semi-skilled	33,516	2.30	2.69	41,826	2.33	2.89
Low skilled	20,098	2.63	1.86	21,979	2.74	2.33
Other	91,803	3.44	4.68	100,163	3.19	4.41

Source: Provincial DCGE model results.

Declines in the labour supply caused by HIV/AIDS are larger than declines in population growth (see Table 5). For example, the population growth rate falls by 1.06% in KZN, while employment growth falls by 1.12%. This reflects the concentration of HIV infections among working age adults. Since employment growth exceeds population growth, the dependency ratio falls slightly from 5.05 to 4.98 under the "No AIDS" scenario. This is driven by African households, whose lower skilled workers have higher prevalence rates and are more affected by HIV/AIDS. Thus, part of African households' higher dependency ratio is driven by HIV/AIDS, which reduces the African working age population faster than the African population as a whole. The reverse is true for other racial groups, albeit only slightly.

High HIV prevalence and larger proportions of AIDS-sick people explain why HIV/AIDS has a more negative effect on labour productivity in KZN than in the rest of the country (see Table 5). Based on observed trends, labour productivity grows at 1.8% under the "AIDS" scenario. However, this is below the 1.92% that would have been achieved in KZN without AIDS-related morbidity and absence from work. Productivity losses from HIV/AIDS are largest for lower skilled African workers due to higher HIV prevalence. These variations in the labour supply and productivity impacts underline the importance of differentiating skill and occupation groups when estimating the macroeconomic impacts of HIV/AIDS.

Based on other studies, we assumed that HIV/AIDS reduces annual TFP growth by 0.5% per year. Overall losses in TFP growth in the DCGE model are slightly larger due to endogenous shifts in resources towards more productive industries (see Table 4). This makes the economy-wide TFP growth rate about 0.6% higher in the "No AIDS" scenario. It should also be noted that the reported changes in the TFP growth rate are independent of the implied TFP changes caused by labour productivity improvements.

Together, higher productivity and labour supply causes an expansion of gross domestic product (GDP). The average annual growth rate in GDP in KZN increases from 2.8% in the "AIDS" scenario to 4.44% in the "No AIDS" scenario (i.e., HIV/AIDS lowers KZN's GDP growth rate by 1.60% per year). This is larger than the negative impact of HIV/AIDS on the rest of South Africa's GDP growth rate, which is reduced by 1.42% per year. Compounding these reductions in annual growth rates means that the KZN and the rest of the South African economies would be 43% and 37% smaller in 2025, respectively, than they could have been were it not for HIV/AIDS.

### Industrial growth

Impacts differ by industry and region (see Table 6). Although the overall decline in economic growth due to HIV/AIDS is larger in KZN than in the rest of South Africa, this is not the case for all individual sectors. The DCGE model captures the varying skill intensities of employment by sector and region from the 2004 Labour Force Survey [17]. This information indicates that the construction industry in KZN is more skill intensive than in the rest of South Africa, with 18% of employment in KZN comprising low-skilled workers compared to 26% in the country as a whole. Thus, by reducing the supply of lower skilled workers, HIV/AIDS hampers the construction industry in the rest of South Africa more than it does in KZN. Similarly, unskilled workers account for 22% of employment in the rest of South Africa's water utilities industry, compared to only 10% in KZN. Therefore, additional GDP growth in these industries is higher in the rest of South Africa than in KZN under the "No AIDS" scenario.

**Table 6:** Change in industrial growth results, 2002-2025

	**Point change in growth rate in "No AIDS" scenario **^1^		Ratio of KZN to Other SA growth rate changes (1)/(2)
		
	KZN	Other SA	
	(1)	(2)	
All sectors (total GDP)	1.60	1.42	1.13
Agriculture	1.88	1.42	1.32
Mining	1.93	1.66	1.16
Food processing	1.74	1.40	1.24
Textiles & clothing	1.66	1.56	1.06
Wood products	1.46	1.46	1.00
Chemicals	1.22	1.47	0.83
Non-metal minerals	1.72	1.70	1.02
Machinery	1.53	1.61	0.95
Electrical machinery	2.28	1.67	1.37
Scientific equipment	1.64	1.41	1.16
Transport equipment	1.59	1.44	1.10
Other manufactures	1.55	1.53	1.01
Electricity	2.05	1.38	1.49
Water and gas	1.47	1.61	0.91
Construction	1.91	1.93	0.99
Trade services	1.82	1.47	1.23
Hotels & catering	1.64	1.45	1.13
Transport services	1.63	1.52	1.08
Communications	1.76	1.51	1.17
Financial services	1.89	1.53	1.24
Business services	1.95	1.49	1.31

Source: Provincial DCGE model results.

1. Point change in annual growth rate between "AIDS" and "No AIDS" scenarios

Although HIV/AIDS has detrimental effects for industries in the rest of South Africa, most of the industries that are most severely hurt are in KZN. This is particularly true for agriculture in KZN, where the AIDS seroprevalence survey data and demographic model predicts especially high HIV prevalence rates. Moreover, this impact on agriculture has negative downstream implications for food processing in KZN. Although the model does not capture rural-urban differences, the large increase in agriculture's growth rate under the "No AIDS" scenario suggests that HIV/AIDS impacts are likely to be more severe in rural areas. Had the model explicitly captured the higher HIV prevalence in rural areas, the outcomes would have been more pronounced.

Of KZN's industries adversely affected by HIV/AIDS, the electrical machinery and electricity industries are most severely undermined. The 2002 supply-use table [18] (on which the DCGE is based) indicates that the electrical machinery sector is less capital intensive than most other industries in the economy. This means that the sector is more vulnerable to the reductions in labour supply caused by HIV/AIDS. Moreover, electrical machinery has a high income elasticity (1.23), which suggests that demand is particularly sensitive to changes in incomes.

By contrast, other light manufacturing industries, such as food products and textiles, have lower income elasticities. As a result, the fall in national income caused by HIV/AIDS generates larger declines in demand for electrical machinery than for food products or textiles. Finally, most jobs in KZN's electrical machinery industry are for lower skilled workers, who are most affected by HIV/AIDS. Together these three characteristics of this industry explain the considerable acceleration of growth in the "No AIDS" scenario.

The water utilities industry in KZN is also less skill intensive than in the rest of South Africa. However, unlike the electrical machinery industry, the water utilities industry is far more capital intensive than most other industries in the economy. Thus, it is not so much the decline in labour supply that undermines growth in this industry, but more the negative consequences of HIV/AIDS for investment and capital accumulation.

Model results indicate that the share of investment in GDP is 2.1% lower under the "AIDS" scenario. While most of this decline in investment is due to the slowdown in economic growth caused by HIV/AIDS, about 28% of the decline results from lower household savings (see Table 4). Thus, the deceleration in economic growth, especially in certain sectors, is driven by the indirect macroeconomic impacts of HIV/AIDS, rather than by its direct impact on population and labour supply.

### Poverty and inequality

**Figure 1:**
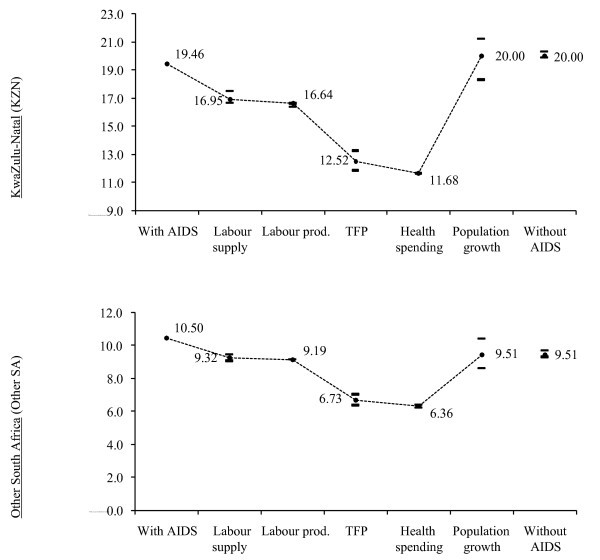
**Channels' impact on final year poverty rate, 2025**. Source: Provincial DCGE model results. Note: Outcomes are cumulative (for example, labour productivity includes the outcomes from labour supply). Horizontal bars show upper and lower bounds after assuming a 20% confidence interval around the additional growth rate resulting from each impact channel.

The impact of HIV/AIDS on income poverty is small (see Table 4). Poverty is measured using the US$2 per day poverty line (which was equal to 161 South African rands per person per month in 2000, the survey year for the micro-simulation module). Model results indicate that without HIV/AIDS, the incidence of poverty (or poverty headcount) would be only slightly lower in the rest of South Africa (i.e., 9.51 under the "No AIDS" scenario compared to 10.50 under the "AIDS" scenario). Moreover, the poverty headcount in KZN would be virtually unchanged (or slightly higher) (see Figure 1).

**Figure 2:**
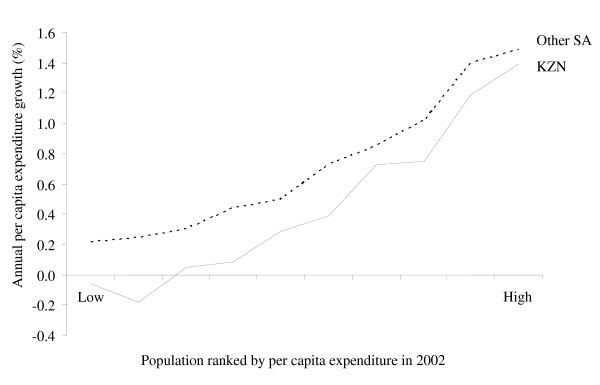
**Regional growth incidence curves, 2002-2025**. Source: Provincial DCGE model results.

The poverty outcomes are extremely sensitive changes in the definition of the poverty line. This is especially true for KZN since its growth incidence curve crosses the x-axis almost at the final year poverty rate (see Figure 2). Greater attention should therefore be paid to the distributional impacts of HIV/AIDS. These impacts are small because the net effect of HIV/AIDS on income poverty depends on two opposing factors. On the one hand, the drop in the working age adult population and the rise in dependency ratios reduce households' incomes. On the other hand, poverty is based on per capita expenditures, which may increase if the decline in household populations exceeds the loss of income. The overall poverty impact therefore depends on which of the two factors dominate.

It is surprising that the model predicts both slightly higher poverty and falling dependency ratios in KZN in the "No AIDS" scenario. We find that poverty remains virtually unchanged because falling wages, caused by labour demand constraints, implies that household incomes rise slower than population growth (see Table 5). Falling wages are more pronounced for lower skilled African workers, whose wage growth rate falls from 2.63% under the "AIDS" scenario to 1.86% under the "No AIDS" scenario.

By contrast, higher skilled workers have lower HIV prevalence rates and these workers, therefore, benefit more from faster economic growth (i.e., their wages rise). Thus, the structural constraints that contribute to high unemployment in the rest of South Africa remain even in the absence of HIV/AIDS. More specifically, the results indicate that KZN and South Africa would continue to become more capital and skill intensive over time, even if the supply and productivity of lower skilled workers were not undermined by HIV/AIDS.

It is also an apparent contradiction that poverty remains virtually unchanged in KZN under the "No AIDS" scenario despite an acceleration of per capita GDP growth by 0.5% (see Table 4). This finding underlines the importance of considering industry and household-level detail that is not captured by aggregate growth models. Aggregate GDP and consumption measures hide the distributional changes caused by HIV/AIDS. Figure 2 shows the "growth incidence curves" for KZN and the rest of South Africa. These curves show the change in the growth rate of annual per capita expenditure for each individual in the population ranked by initial expenditure levels.

The mean of both regions' curves is positive, reflecting the increase in aggregate per capita incomes in the "No AIDS" scenario. However, the fact that the growth incidence curves are upward sloping means that lower income households benefit less than higher income households in the "No AIDS" scenario. This suggests that income inequality would increase between 2002 and 2025 if HIV/AIDS were eliminated.

A number of reasons explain this result. First, as mentioned earlier, the increased supply of lower skilled workers is offset by falling wages, leaving per capita incomes among households at the lower end of the distribution largely unchanged. The reverse is true for higher skilled workers whose wages rise with faster economic growth. Secondly, unemployment is high among working age adults living in poorer households. Therefore, reducing adult mortality may not reduce these households' dependency ratios, causing per capita incomes to fall. This is the case for lower income households in KZN, whose growth incidence curve is negative. While removing the effects of HIV/AIDS improves overall household welfare, it is detrimental for lower income household poverty in KZN, where unemployment is especially severe.

A third reason for the increase in inequality is shown by measuring the contribution of the five impact channels to overall changes in GDP growth rates and poverty rates under the "No AIDS" scenario (see Table 7). The decomposition was conducted by only imposing single impact channels on the DCGE model. This is a reasonable approximation of each channels' contribution, although it may exclude interactions between channels when they are jointly imposed. The table shows that the effect of HIV/AIDS on labour supply and TFP dominates growth outcomes (i.e., 85% of the increase in the GDP growth rate). The finding that TFP losses from HIV/AIDS cause almost a one percent drop in economic growth is consistent with the findings of other studies [19-21].

**Table 7:** Contributions of impact channels, 2002-2025

	Growth rate (%)		Poverty rate (%-point)	
	
	KZN	Other SA	KZN	Other SA
Total change	1.60	1.42	0.54	-0.99
Labour supply	0.63	0.50	-2.51	-1.36
Labour productivity	0.11	0.08	-0.31	-0.32
Total factor productivity	0.73	0.73	-4.13	-2.64
Private savings/investment	0.13	0.11	-0.84	-0.56
Population growth	0.00	0.00	8.33	3.88

Source: Provincial DCGE model results.

We have already discussed how increases in labour supply cause declines in lower skilled workers' wages, thus reducing the income gains from reduced mortality in the "No AIDS" scenario. Moreover, increased labour productivity and reduced health spending have only small effects on economic growth. Thus, the direct channels linking HIV/AIDS to poorer households are less important than the indirect TFP effects.

The dominance of indirect impact channels is also evident in the poverty decomposition, which shows how the direct channels' contributions to poverty reduction are smaller than that of TFP growth. They are also far smaller than the downward pressure placed on per capita incomes by higher population growth. Thus it is TFP that drives the overall increase in growth and reduction in poverty in the rest of South Africa under the "No AIDS" scenario. However, TFP growth does not just benefit households with HIV-infected working adults. Rather, faster economic growth driven by TFP improvements drives up demand for all workers, including those whose HIV prevalence is initially low.

Thus, the third reason why removing HIV/AIDS causes inequality to rise is that TFP benefits all households and workers regardless of whether they are infected by HIV. Higher income households therefore benefit from faster economic growth despite low infection rates. This finding highlights the importance of taking macroeconomic spillovers into account when assessing the overall impact of HIV/AIDS on growth and poverty.

## Conclusion

KwaZulu-Natal, together with the rest of South Africa, suffers from severe unemployment and poverty. Moreover, the province has one of the highest HIV prevalence rates in the world. This paper has estimated the impact of HIV/AIDS on economic growth and income poverty in KZN and the rest of South Africa. Drawing on the findings from a firm-level survey in four of KZN's major economic sectors, we integrated the projections from a demographic model within a regionalized DCGE model. This in turn was linked to a survey-based micro-simulation module in order to estimate poverty and distributional outcomes.

This approach extends previous studies by: focusing on South Africa's most afflicted region; basing its projections on more reliable estimates of HIV prevalence for workers across occupational groups; and explicitly integrating demographic, economy-wide and survey-based models.

The results indicate that HIV/AIDS undermines economic growth in South Africa. It lowers the GDP growth rate by 1.60% and 1.42% per year in KZN and the rest of South Africa, respectively. Cumulating these losses means that the KZN economy would be 43% smaller in 2025 than it would be in the absence of HIV/AIDS. The rest of the country's economy is similarly 37% smaller. While the detrimental growth effect is large, the impact of HIV/AIDS on the regional poverty headcounts is relatively small, and that inequality would be higher in the absence of HIV/AIDS.

The small change in per capita incomes among the poor population should be interpreted alongside the 11.8 million who are projected to die as a result of HIV/AIDS between 2002 and 2025. Thus, the gains in economic growth in the absence of HIV/AIDS are sufficient to offset the pressure placed on poverty by a substantially larger population. Moreover, the incentive to mitigate the effects of HIV/AIDS lies not only with poorer households and those with infected members, but also with the uninfected and higher income households, who stand to benefit from faster economic growth and rising incomes. These findings reveal the significant burden that HIV/AIDS places on future economic development in KwaZulu-Natal and the rest of the South Africa, and underlines the need for policies and investments to curb the pandemic.

## Competing interests

The authors declare that they have no competing interests.

## Authors' contributions

JT developed the macroeconomic model, collected data, ran the simulations, produced the output tables and figures, and drafted the manuscript, JG conceived of the study and participated in its design and coordination and edited the manuscript, and GG participated in the study design and coordination. All authors have read and approved the final version of the manuscript.

## Supplementary Material

Additional file 1 **Simplified CGE model variables, parameters and equations**. The information provided outlines the structure of the CGE model, its variables, parameters and equations Click here for file
