# Supplementary material for: HIV/AIDS, growth and poverty in KwaZulu-Natal and South Africa: an integrated survey, demographic and economy-wide analysis
Source: J Int AIDS Soc. 2009 Sep 16;12:18. doi: 10.1186/1758-2652-12-18 (PMC2754430; doi:10.1186/1758-2652-12-18)
Supplement: Additional file 1 — Simplified CGE model variables, parameters and equations. The information provided outlines the structure of the CGE model, its variables, parameters and equations [file 1758-2652-12-18-S1.DOC]

**Additional File 1: Simplified CGE model equations, variables and parameters**

| Static model equations | | | |
| --- | --- | --- | --- |
| Production | 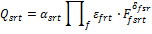 | | (1) |
| Factor returns | 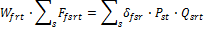 | | (2) |
| Income | 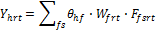 | | (3) |
| Consumption | 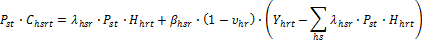 | | (4) |
| Investment | 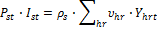 | | (5) |
| Labour market | 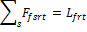 | *f is labour* | (6) |
| Capital market | 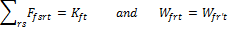 | *f is capital* | (7) |
| Product market | 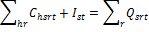 | | (8) |
| Dynamic equations and links to the demographic model | | |  |
| Population | 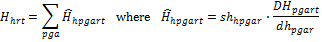 | | (9) |
| Labour supply | 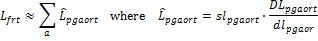 | *f is labour* | (10) |
| Labour productivity | 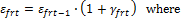 |  |  |
|  | 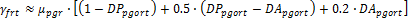 | *f is labour* | (11) |
| Technical change | 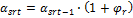 | | (12) |
| Capital supply | 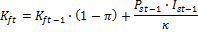 | *f is capital* | (13) |
|  |  |  |  |

**Additional File 1 continued:**

|  |  | | |  |  | | |
| --- | --- | --- | --- | --- | --- | --- | --- |
| Subscripts | | | | Endogenous variables in CGE model | | | |
| *f* | | Factors | | *C* | | Household consumption demand quantity | |
| *r* | | Regions | | *F* | | Factor demand quantity | |
| *s* | | Sectors | | *I* | | Investment demand quantity | |
| *t* | | Time periods | | *K* | | National capital supply | |
| *p* | | Population group (race) | | *L* | | Regional labour supply | |
| *g* | | Gender | | *M* | | Migration rate between regions r and r' | |
| *a* | | Age cohort | | *P* | | Commodity price | |
| *o* | | Occupation group | | *Q* | | Output quantity | |
| Exogenous parameters | | |  | *W* | | Average factor return | |
| α | | Total factor productivity (production shifter) | | *Y* | | Total household income | |
| β | | Household marginal budget share | | Projections from demographic model | | |  |
| γ | | Factor-specific productivity growth rate | | *DH* | | Population projection | |
| δ | | Factor input share parameter | | *DL* | | Labour supply projection | |
| ε | | Factor-specific productivity (input shifter) | | *DP* | | Predicted HIV prevalence rate | |
| θ | | Household share of factor income | | *DA* | | Predicted full-blown AIDS prevalence rate | |
| κ | | Base price per unit of capital stock | | Base-year (2002) stock estimates | | |  |
| λ | | Per capita subsistence consumption quantity | | *sh* | | Household population profile (household survey) | |
| φ | | Hick’s neutral productivity growth rate | | *sl* | | Labour force profile (labour survey) | |
| π | | Capital depreciation rate | | *dh* | | Population profile (demographic model) | |
| ρ | | Investment commodity expenditure share | | *dl* | | Labour force profile (demographic model) | |
| σ | | Exogenous factor supply growth rate | |  | |  | |
| υ | | Household marginal propensity to save | |  | |  | |
| μ | | Exogenous labour productivity growth rate | |  | |  | |
|  | |  | |  | |  | |
